# Proteomics of stress-induced cardiomyopathy: insights from differential expression, protein interaction networks, and functional pathway enrichment in an isoproterenol-induced TTC mouse model

**DOI:** 10.7717/peerj.18984

**Published:** 2025-02-13

**Authors:** Liuyang Tian, Botao Liu, Ying Ren, Jian Cui, Zhihua Pang

**Affiliations:** 1 Interventional Center of Valvular Heart Disease, Beijing Anzhen Hospital, Beijing, China; 2 China Medical University, Shenyang, China; 3 Department of Cardiology, Tianjin Union Medical Center, Tianjin, China; 4 Department of Cardiology, The First Affiliated Hospital of Nankai University, Tianjin, China

**Keywords:** Takotsubo cardiomyopathy, Stress-induced cardiomyopathy, Proteomics, Isoproterenol

## Abstract

**Backgrounds:**

Takotsubo cardiomyopathy (TTC), also known as stress-induced cardiomyopathy, is a condition characterized by transient left ventricular dysfunction without coronary artery obstruction.

**Methods:**

We utilized label-free quantitative proteomics to analyze protein expression in a murine model of TTC, induced by a high dose of isoproterenol (ISO) injection.

**Results:**

We found that a single high dose of ISO injection in mice could induce stress-related cardiac dysfunction.The proteomic analysis revealed 81 differentially expressed proteins (DEPs) between the ISO and control groups—39 were upregulated, and 42 were downregulated. Key pathways enriched by Gene Ontology (GO) analysis included collagen fibril organization, cholesterol biosynthesis, and elastic fiber assembly. Kyoto Encyclopedia of Genes and Genomes (KEGG) pathway enrichment indicated significant changes in unsaturated fatty acid biosynthesis, glutathione metabolism, steroid biosynthesis, and ferroptosis. Key hub proteins identified by the protein-protein interaction (PPI) network included Ntrk2, Fdft1, Serpine1, and Cyp1a1. Gene set enrichment analysis (GSEA) showed upregulation in terpenoid backbone biosynthesis, oxidative phosphorylation, and ferroptosis, with downregulation in pathways such as systemic lupus erythematosus and Rap1 signaling.

**Conclusions:**

This study employed high-throughput liquid chromatography-tandem mass spectrometry (LC-MS/MS) to identify key proteins associated with energy metabolism, oxidative stress, inflammation, and cell death in TTC. These findings provide new insights into the molecular mechanisms of stress-induced myocardial injury and may offer potential therapeutic targets for mitigating cardiovascular damage under stress conditions.

## Highlights

- Isoproterenol induces stress-related cardiac dysfunction in a Takotsubo cardiomyopathy (TTC) mouse model, providing a robust platform for studying stress-induced myocardial injury.
- Proteomic analysis reveals crucial pathways, including ferroptosis, oxidative phosphorylation, and collagen fibril organization, which are closely associated with energy metabolism, oxidative stress, and cardiac structural remodeling.
  Key hub proteins such as Ntrk2, Fdft1, and Serpine1 regulate critical biological processes, offering novel insights and potential therapeutic targets for mitigating stress-induced myocardial damage.

## Introduction

Stress-induced myocardial injury, also termed stress cardiomyopathy, is often characterized by temporary heart dysfunction, structural damage, and in severe cases, acute heart failure (Boland, Lee & Bleck, 2015). Takotsubo cardiomyopathy (TC), also known as stress-induced cardiomyopathy or “broken heart syndrome”, is characterized by transient left ventricular dysfunction that often mimics acute myocardial infarction (AMI) but occurs without obstructive coronary artery disease (Hayashi et al., 2023). First described in Japan, TC is typically precipitated by physical or emotional stress, leading to acute chest pain and ECG changes that resemble those of a heart attack (Amin, Amin & Pradipta, 2020).

Previous studies have implicated neurohormonal factors, such as catecholamines (de Carvalho et al., 2021), are associated with the pathophysiology of stress-related myocardial dysfunction. This damage may result from excessive stimulation of β-adrenergic receptors, leading to intracellular calcium overload, oxidative stress, and subsequent myocardial stunning (Adzika et al., 2021). Furthermore, catecholamine toxicity has been linked to microvascular dysfunction, impaired coronary blood flow, and energy metabolism abnormalities (Citro et al., 2023), all of which contribute to the observed ventricular dysfunction. However, the specific molecular events, particularly at the proteome level, remain inadequately defined.

Recent advances in proteomics have enabled large-scale identification and quantification of proteins and post-translational modifications, providing insights into the mechanisms underlying cellular (An et al., 2020). By profiling protein expression changes in myocardial tissues under acute stress, we aim to better understand the molecular basis of stress-induced myocardial damage (Quan & Liu, 2024).

Isoproterenol (ISO) is a synthetic catecholamine that acts as a potent β-adrenergic receptor agonist. Previous studies have demonstrated that ISO administration can reliably reproduce the acute cardiac dysfunction and pathological features observed in TC, making it a widely used model to study stress-induced cardiomyopathy (Yoganathan et al., 2023). Following the induction of the condition, we employed LC-MS/MS-based proteomics to investigate the effects of acute stress on the myocardial proteome in a mouse model, highlighting potential pathways and biomarkers involved in stress-induced cardiac injury.

Thus, the objectives of this study were to (1) establish a Takotsubo cardiomyopathy model in mice *via* ISO injection, (2) perform proteomic analysis of cardiac tissue to identify DEPs, and (3) conduct bioinformatic analyses, including GO and KEGG pathway enrichment, protein-protein interaction (PPI) network analysis, and gene set enrichment analysis (GSEA), to uncover the underlying molecular mechanisms of TC.

## Materials and Methods

### Animals

Experiments were approved by the ethical policies and procedures approved by the ethics committee of the Tianjin union medical center (Approval no. 2018C03). Adult female C57BL/6J mice (8 weeks old, *n* = 30) were randomly divided into two groups: the control group (Con) and the stress-induced group (ISO injection, ISO).

The mice were housed in a temperature- and humidity-controlled environment, with the room maintained at 22 ± 1 °C and 50 ± 10% relative humidity, with a 12-h light/dark cycle and free access to food and water. Thirty adult female C57BL/6J mice (8 weeks old) were obtained from Sipeifu biotechnology company (Beijing, China). Mice were administered sodium pentobarbital (30 mg/kg) *via* intraperitoneal injection to induce deep anesthesia. Once confirmed to be fully anesthetized, cervical dislocation was performed.

### Mouse stress-induced model

A single high dose of isoproterenol (ISO) has been shown to induce stress-related responses in rodent models (Shao et al., 2013; Liao et al., 2022). A single high dose of ISO has been shown to induce stress-related responses in rodent models (Walsh-Wilkinson, Arsenault & Couet, 2021), with females being more susceptible to stress-induced myocardial injury. Based on this, we selected female mice as the subjects for constructing the stress-induced model. According to the previous studies (Liao et al., 2022), each female mice were administered an intraperitoneal injection of ISO (Sigma-Aldrich, St. Louis, MO, USA) at a dose of 200 mg/kg to establish the Stress-Induced Model. Control mice were injected with an equivalent volume of saline. After 1 day, 3 days, and 7 days, mice were sacrificed, and cardiac tissues were collected for proteomic and subsequent analyses.

### Echocardiography

Echocardiography was performed at multiple time points: before ISO injection, and on days 1, 3, and 7 post-injection. For these studies, five biological replicates were selected per group for the control group and the ISO-injected groups at each time point.

M-mode and 2-D echocardiographic studies were performed using a VisualSonics Vevo 2,100 high-resolution ultrasound system (FUJIFILM VisualSonics, Toronto, Canada). A linear array transducer with a frequency of 9 MHz was used to conduct the cardiac function assessment. The transducer was positioned on the left chest of the mouse to obtain satisfactory two-dimensional long-axis parasternal images of the left ventricle. Left ventricular end-systolic volume (LVESV) and left ventricular end-diastolic volume (LVEDV) were measured. The left ventricular ejection fraction (EF, %) was calculated using the CUBED formula method. For each parameter, the mean value of three consecutive cardiac cycles was used. All echocardiography procedures and data analyses were conducted by a dedicated, trained operator.

### Hematoxylin and eosin staining

The hearts were fixed in 4% paraformaldehyde for 24–48 h at room temperature. Then, the tissues were dehydrated through a graded series of ethanol, cleared in xylene, and embedded in paraffin. Paraffin-embedded heart tissues were sectioned into 4–5 µm thick slices using a microtome and mounted onto glass slides. The sections were deparaffinized in xylene and rehydrated through decreasing concentrations of ethanol. Subsequently, the tissue sections were stained with hematoxylin (51275; Sigma-Aldrich, St Louis, MO, US) for 5–10 min to visualize nuclei, followed by washing in running tap water. They were then counterstained with eosin (318906; Sigma-Aldrich, St Louis, MO, US) for 1–3 min to stain the cytoplasm and extracellular matrix. After staining, the sections were dehydrated through graded ethanol, cleared in xylene, and coverslipped using a permanent mounting medium. The slides were examined under a light microscope for histopathological analysis. The histopathological analysis was performed at multiple time points: before ISO injection, and on days 1, 3, and 7 post-injection. For these studies, five biological replicates were selected per group for the control group and the ISO-injected groups at each time point. The scoring standardes were defined as follows: a score of 0 represented no detectable damage, one corresponded to damage involving less than 25% of the tissue area, two indicated damage affecting 25–50% of the tissue, three was assigned to damage involving 50–75% of the area, and four represented damage affecting over 75% of the tissue.

### Protein extraction and digestion

Proteomic analysis was performed for two groups: the control group (Con) and the stress-induced group (ISO injection for 7 days). For this analysis, five biological replicates per group were used to ensure robust and reproducible findings.

Myocardial tissue samples (~50 mg) were homogenized in the lysis buffer containing protease inhibitors to prevent protein degradation. After centrifugation, the supernatants were collected, and protein concentrations were determined using the Bradford assay.

### LC-MS/MS analysis

Liquid chromatography-tandem mass spectrometry (LC-MS) was carried out as described previously (Goldman et al., 2019). Tryptic peptides were separated using liquid chromatography on a C18 column and analyzed by tandem mass spectrometry on a Q-Exactive Orbitrap mass spectrometer (Thermo Fisher Scientific, Waltham, MA, USA). Raw MS data were processed using MaxQuant software (version 1.6.10.4) for protein identification and quantification. A minimum of two unique peptides was required for confident protein identification. The Label-Free Quantification (LFQ) algorithm in MaxQuant was used for protein quantification. Missing values in the proteomics dataset were imputed to address sparsity in the LFQ data. Specifically, missing values were replaced with values drawn from a normal distribution centered around the lower detection limit of the dataset, as implemented in the MaxQuant software pipeline. The proteomic data were then analyzed for differential expression between control and stress-induced models. The analysis of differentially expressed proteins (DEPs) was implemented through the Limma package in the R language. The DEPs are defined as |logFC| > 1.5 and *p*-value < 0.05.

Heat maps and volcano plots were used to visualize the differentially expressed proteins (DEPs) identified in the study. For the heat map, normalized expression values of DEPs (|logFC| > 1.5 and *p*-value < 0.05) were used, and hierarchical clustering was applied to group proteins and samples based on expression patterns. The volcano plot displayed log2 fold changes (x-axis) against -log10(*p*-values) (y-axis), with significantly upregulated proteins highlighted in red and downregulated proteins in blue. Both visualizations were generated using R software (version 4.3.1) with the pheatmap and ggplot2 packages.

### Bioinformatics and pathway analysis

Differentially expressed proteins were subjected to Gene Ontology (GO) and Kyoto Encyclopedia of Genes and Genomes (KEGG) pathway enrichment analysis to identify relevant biological processes and signaling pathways. GO annotation of the proteome was performed based on data from the UniProt-GOA database (http://www.ebi.ac.uk/GOA/). For pathway analysis, proteins were annotated using the KEGG database (Kanehisa et al., 2021). Protein pathway annotation was conducted using the KEGG Automatic Annotation Server (KAAS) to assign KEGG Orthology (KO) identifiers and retrieve pathway descriptions. The resulting annotations were then mapped to pathways using the KEGG Mapper tool.

### Protein-protein interaction network construction

A protein-protein interaction (PPI) network was constructed using the STRING database (version 11.5) (Szklarczyk et al., 2023) to visualize the interactions between differentially expressed proteins (confidence score threshold = 0.7). The network was visualized using Cytoscape software (version 3.9.1) (Doncheva et al., 2023), and top hub proteins were identified based on degree centrality, which quantifies the number of direct interactions each protein has in the network.

### Gene set enrichment analysis

Differentially expressed proteins were subjected to GSEA to identify significantly enriched biological pathways between the AMI and Con groups. GSEA was performed using the KEGG and Reactome databases (Fang, Liu & Peltz, 2023), with normalized enrichment scores (NES), *p*-values, and false discovery rates (FDR) used to assess pathway significance. Pathways with an FDR < 0.05 were considered significantly enriched.

### Quantitative PCR experiment

Quantitative PCR (qPCR) was performed to validate the mRNA expression of the top 20 differentially expressed genes, using three biological replicates per group to confirm the key proteomic results. Total RNA was extracted from cardiacs using TRIzol reagent (TRIzolTM (15596026); ThermoFisher Scientific, Waltham, MA, USA), following the manufacturer’s protocol. The quality and concentration of RNA were assessed using spectrophotometry. Subsequently, the extracted total RNA was reverse-transcribed into complementary DNA (cDNA) using the GoTaq qPCR Master Mix (A6001; Promega, Madison, WI, USA). The resulting cDNA was then subjected to quantitative real-time PCR (qPCR) using a Bio-Rad thermal cycler (Bio-Rad, Hercules, CA, USA). The relative expression levels of each gene were calculated using the 2^(−ΔΔCt) method, with GAPDH as the endogenous control. Each sample was run in triplicate, and statistical significance was determined using a Student’s t-test, with *p* < 0.05 considered significant. The sequences of these primers are provided in Table S1.

### Western blotting

Western blotting was performed to validate the protein expression of key hub proteins identified from the PPI network, which were also part of the DEPs. For this analysis, three biological replicates per group were used to confirm the key proteomic findings.

Protein lysates were extracted from the samples using RIPA buffer supplemented with phenylmethylsulfonyl fluoride (PMSF). Protein concentrations were determined using the BCA assay. For each condition, 20 micrograms of protein were loaded onto 4–12% SDS-polyacrylamide gels and separated by electrophoresis. The proteins were subsequently transferred onto nitrocellulose membranes for analysis.

The membranes were blocked with 5% milk for 2 h and incubated overnight at 4 °C with primary antibodies specific to the proteins of interest. The following primary antibodies were used: Ntrk2 (13129-1-AP, 1:2,000), Fdft1 (13128-1-AP, 1:2,000), Serpine1 (13801-1-AP, 1:1,000), and Cyp1a1 (13241-1-AP, 1:2,000), all purchased from Proteintech (USA). Beta-actin (ACTB, ab6276, 1:10000) was used as a loading control and obtained from Abcam (Cambridge, UK).

After washing the membranes three times, they were incubated with appropriate secondary antibodies. Protein bands were visualized using the Amersham Imager 600 system (GE Healthcare Life Sciences, Chicago, IL, USA). Relative protein levels were quantified using ImageJ software (Madison, WI, USA) and normalized to ACTB as the control.

### Statistical analysis

All statistical analyses were conducted using GraphPad Prism. Results from the proteomic analysis were presented as fold changes and differential expression was considered significant at a threshold of |logFC| > 1.5 and *p* < 0.05. For comparisons involving multiple groups (*e.g*., echocardiography and histopathological scoring), statistical significance was determined using one-way ANOVA followed by Tukey’s *post hoc* test. For qPCR and WB validation, relative mRNA and protein expression levels were analyzed using a two-tailed Student’s t-test. Data are presented as mean ± standard deviation (SD). Statistical significance was denoted as *p < 0.05, **p < 0.01, and ***p < 0.001. The number of biological replicates used in each analysis is indicated in the corresponding figure legends.

## Results

### ISO-induced TTC-like cardiomyopathy in female mice

**Figure 1:**
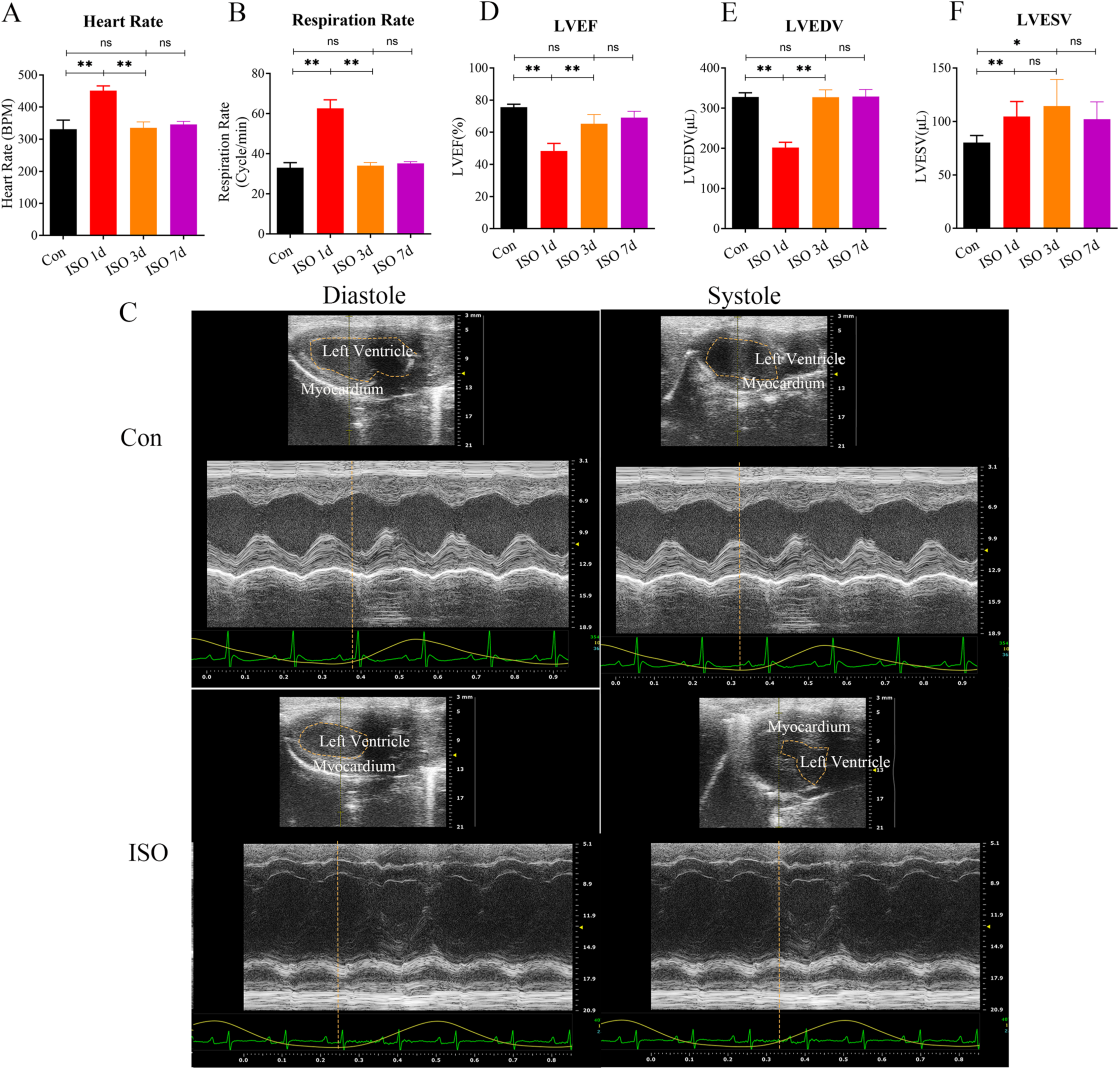
Echocardiographic assessment of ISO-induced TTC-like cardiomyopathy in female mice. (A) Heart rate (beats per minute, bpm) measured at baseline (before ISO injection) and 1, 3, and 7 days post-injection. (B) Respiratory rate (breaths per minute, bpm) measured at the same time points. (C) Representative echocardiographic images demonstrating apical ballooning in ISO-treated mice compared to the control group. (D) Left ventricular ejection fraction (LVEF, %), (E) left ventricular end-diastolic volume (LVEDV, µL), and (F) left ventricular end-systolic volume (LVESV, µL) measured at the same time points. Data are presented as mean ± SD from 5 animals per group at each time point. Statistical significance was determined using one-way ANOVA followed by Tukey’s *post hoc* test (ns, not significant; *p < 0.05, **p < 0.01, compared to baseline or control group).

In this study, we investigated the development of TTS-like cardiomyopathy in female mice following a single injection of ISO. 1 day after ISO administration, a noticeable increase in both heart rate and respiratory rate was observed in the mice (Figs. 1A–1B), indicating an acute stress response. This heightened activity peaked 1 day after the injection but gradually returned to near baseline levels by 3 and 7 days post-injection (Figs. 1A–1B).

We confirmed the presence of left ventricular apical ballooning (Fig. 1C), a hallmark feature of TTS, which mimics the clinical presentation of Takotsubo cardiomyopathy in humans.

To evaluate the TTS-like responses, left ventricular function was monitored by measuring the left ventricular ejection fraction (LVEF), along with left ventricular end-systolic volume (LVESV) and left ventricular end-diastolic volume (LVEDV). The results showed that ISO-treated mice exhibited significant acute cardiac dysfunction one-day post-injection, characterized by a marked reduction in LVEF, LVESV, and LVEDV (Figs. 1D–1F). These changes indicate a substantial impairment in left ventricular systolic function following ISO injection.

Over the following days, the cardiac function in the female mice gradually improved, with LVEF nearly returning to baseline levels and LVESV and LVEDV also normalizing by day 7 post-injection (Figs. 1D–1F). These findings suggest that ISO injection induced transient systolic dysfunction, with significant recovery over time.

**Figure 2:**
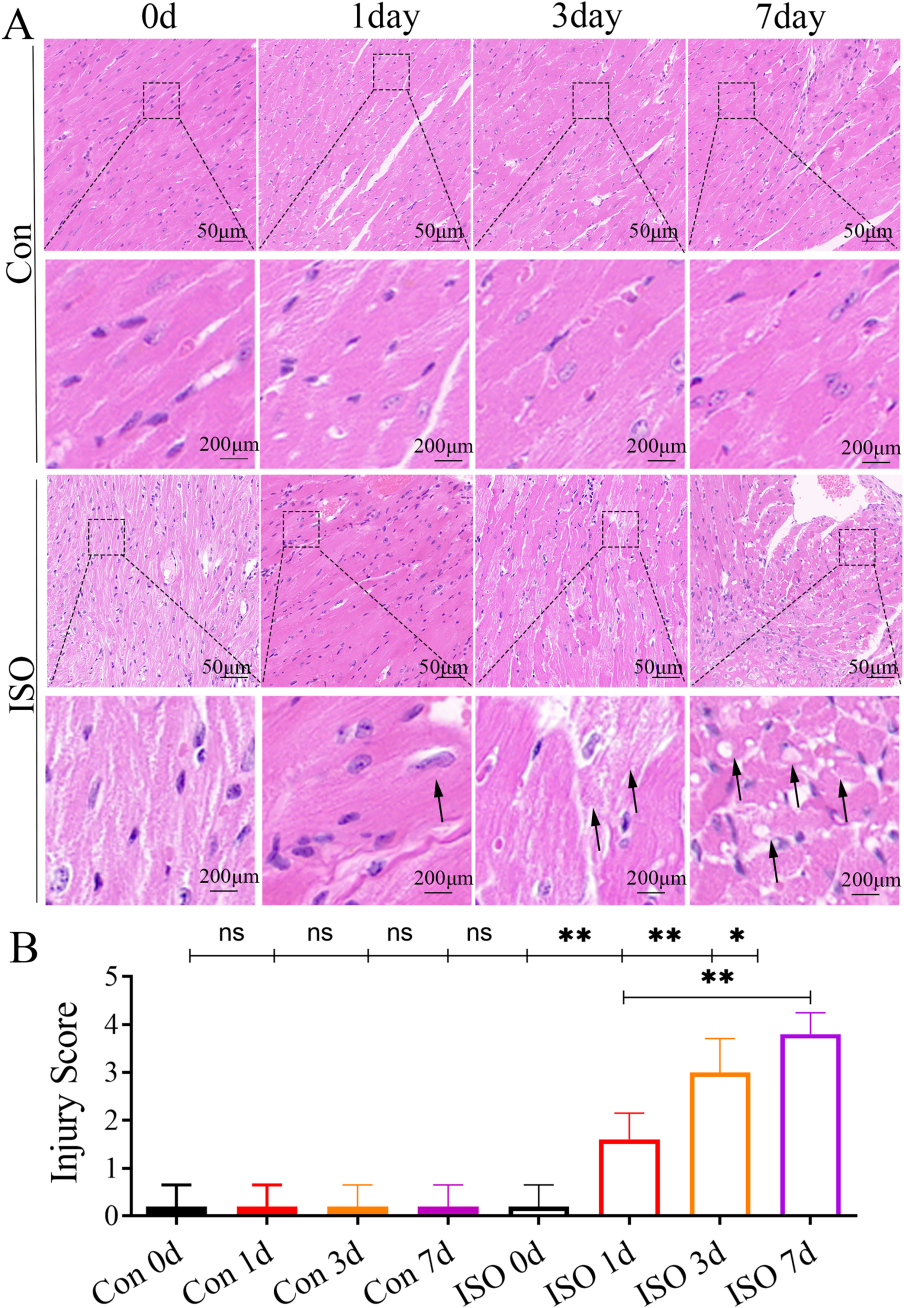
Histopathological changes in myocardial tissues from ISO-induced TTC-like cardiomyopathy. (A) Hematoxylin and eosin (H & E) staining of myocardial tissue at baseline (control), and 1, 3, and 7 days after ISO injection. Representative images show progressive myocardial cell thickening, dissolution, and structural disorganization. Scale bar = 50 µm. (B) Quantitative scoring of myocardial damage based on injury area, defined as: 0 = no damage, 1 = <25%, 2 = 25–50%, 3 = 50–75%, and 4 = >75% damage. Data represent mean ± SD from five animals per group at each time point. Statistical significance was determined using one-way ANOVA followed by Tukey’s *post hoc* test (ns, not significant; *p < 0.05, **p < 0.01 compared to baseline or control group).

At 1 and 3 days following ISO injection, there was a slight increase in the thickness of myocardial cells, these changes were not particularly prominent. By 7 days post-injection, we observed a marked thickening of the myocardial cell bundles along with more pronounced cellular dissolution (Fig. 2A). Statistical analysis of the injury scores revealed a progressive increase in myocardial damage over time following ISO injection, with significant differences observed (Fig. 2B). These findings suggest that the ISO-induced acute cardiac dysfunction preceded the development of visible cardiac remodeling and structural damage.

### Differentially expressed proteins in myocardial tissue

**Figure 3:**
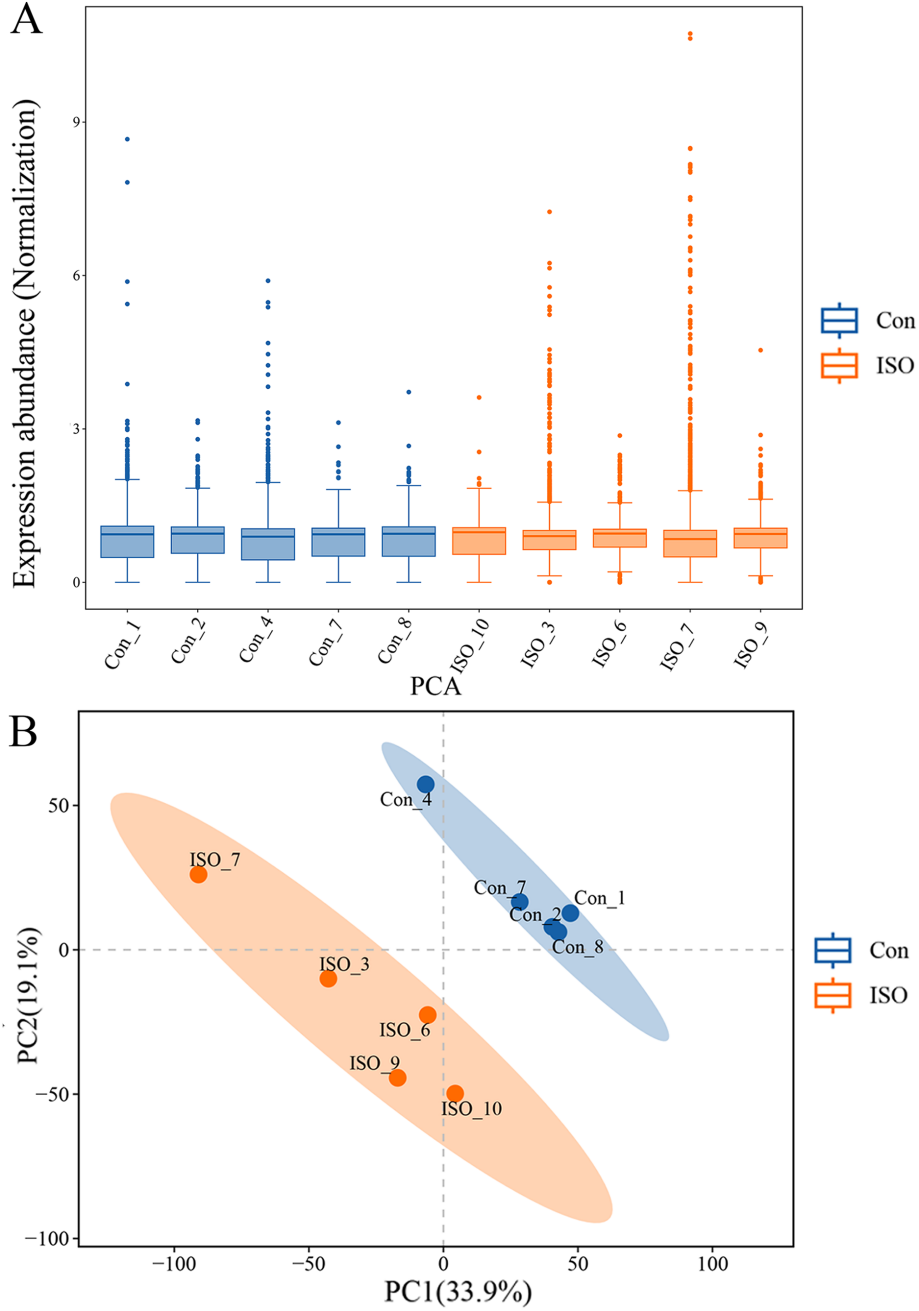
Proteomic profiling of myocardial tissues from ISO-induced and control groups. (A) Boxplot of protein expression levels in myocardial tissues from control and ISO-treated mice. Data represent log-transformed normalized protein intensities from five biological replicates per group. (B) Principal component analysis (PCA) of protein expression data showing distinct clustering of control and ISO-treated samples, indicating significant differences in proteomic profiles.

To further investigate the molecular mechanisms underlying ISO-induced TTC, we conducted a label-free quantitative proteomics analysis of myocardial tissue from ISO-injected mice. LC-MS/MS analysis identified a total of 6,463 proteins in myocardial tissues from both control and ISO-treated mice. A boxplot of protein expression levels (Fig. 3A) was generated to provide an overview of the distribution and consistency of detected protein abundances across samples. Principal component analysis (PCA) was performed using the expression levels of these proteins to visualize the relationship between samples across different dimensions. The PCA analysis indicated a clear separation between the control and ISO-treated groups (Fig. 3B), suggesting significant differences in protein expression profiles between the two groups.

**Figure 4:**
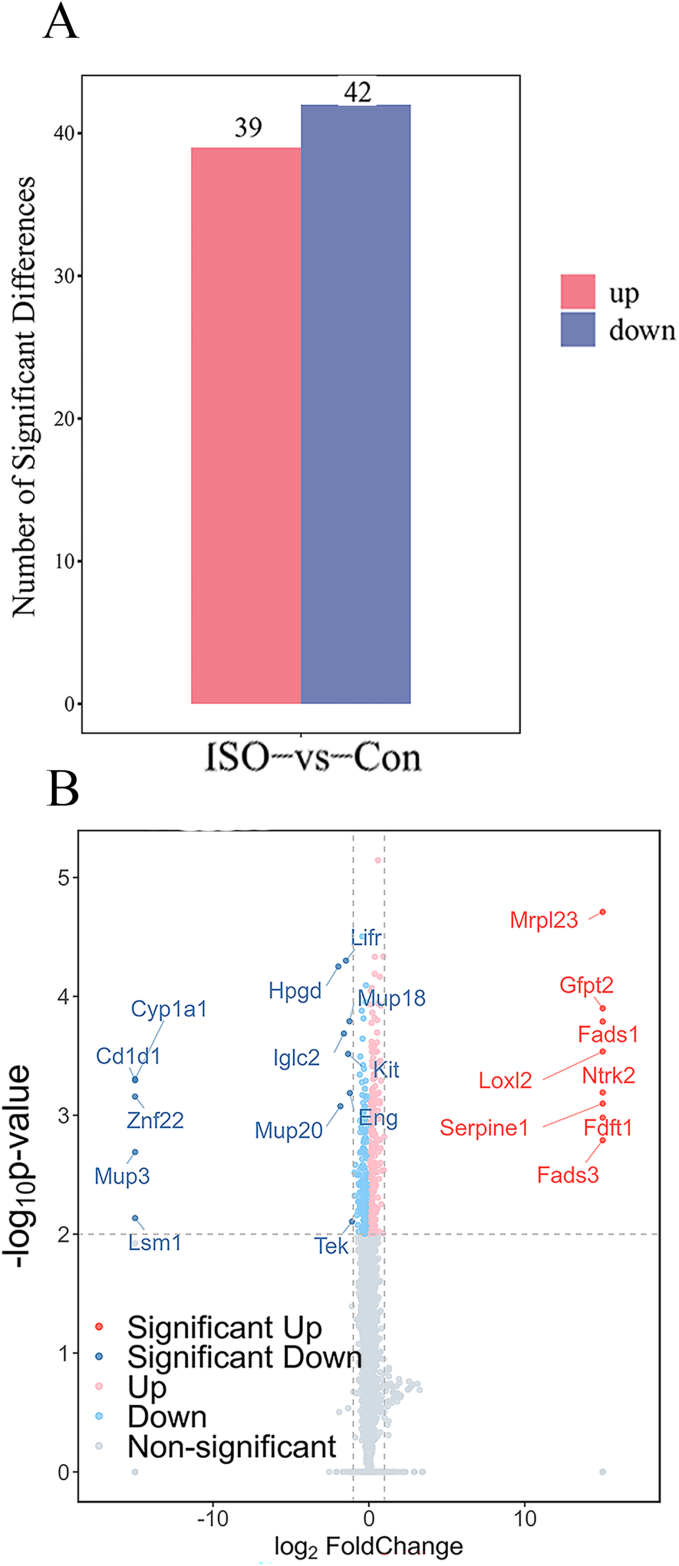
Identification and validation of differentially expressed proteins (DEPs) between ISO-treated and control groups. (A) Total number of DEPs identified (81 proteins) with 39 upregulated (red) and 42 downregulated (blue) proteins (|log2FC| ≥ 1.5, *p* < 0.05). (B) Volcano plot displaying the significance (−log10 *p*-value) and fold change (log2FC) of all detected proteins. Upregulated proteins are shown in red, downregulated proteins in blue, and non-significant proteins in gray.

To identify proteins significantly affected by acute stress, differential expression analysis was performed, resulting in the identification of 81 differentially expressed proteins (DEPs) with a cutoff of *p* < 0.05 and |logFC| ≥ 1.5. Among these, 39 proteins were upregulated, while 42 proteins were downregulated (Fig. 4A). A volcano plot and heatmap were generated (Figs. 4B, S1) to visually display these differentially expressed proteins, with distinct clusters of upregulated and downregulated proteins in response to ISO treatment. The top five upregulated proteins included: Mrpl23, Gfpt2, Fads1, Loxl2, and Ntrk2. The top five downregulated proteins were: Cyp1a1, Cd1d1, Znf22, Mup3, and Lsm1. The full list of DEPs is provided in Table S2 for further reference and detailed analysis.

### GO and KEGG functional enrichment analysis of DEPs

**Figure 5:**
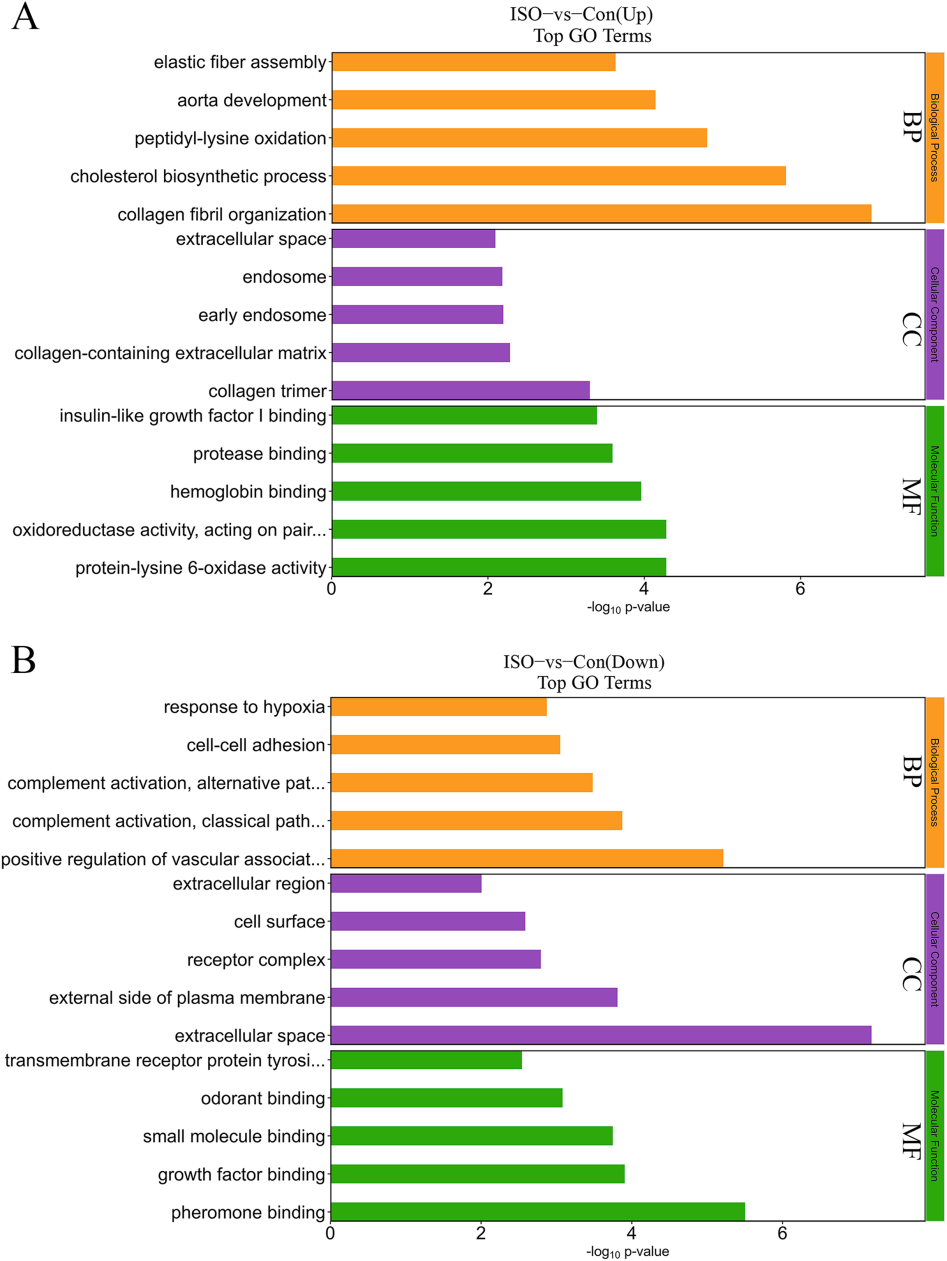
Gene Ontology (GO) enrichment analysis of differentially expressed proteins (DEPs). (A) GO analysis of upregulated DEPs reveals significant enrichment in pathways related to collagen fibril organization, cholesterol biosynthesis, and elastic fiber assembly. (B) Downregulated DEPs are significantly enriched in pathways including positive regulation of vascular smooth muscle differentiation, complement activation (classical pathway), and response to hypoxia. Data are derived from five biological replicates per group, with *p* < 0.05 considered significant.

To further elucidate the biological significance of the DEPs following ISO-induced TTC, we performed GO enrichment analysis and KEGG pathway analysis. These analyses provided insights into the affected biological processes, cellular components, molecular functions, and pathways influenced by acute stress. The top enriched processes in the upregulated DEPs included: collagen fibril organization, cholesterol biosynthetic process, peptidyl-lysine oxidation, aorta development, and elastic fiber assembly (Fig. 5A). These findings suggest that ISO treatment may lead to collagen reorganization and contribute to cardiac fibrosis, which is a known feature of myocardial remodeling in TTC. Additionally, alterations in cholesterol biosynthesis and elastic fiber assembly imply changes in the mechanical properties and elasticity of the myocardium, potentially influencing cardiac function and structure under stress. Conversely, the DEPs in the downregulated pathways were significantly enriched in: positive regulation of vascular-associated smooth muscle cell differentiation, complement activation, classical pathway, cell-cell adhesion, and response to hypoxia (Fig. 5B). The downregulation of these processes indicates that ISO treatment may impair vascular smooth muscle contraction and relaxation, which could impact vascular function and cardiac perfusion. Additionally, the inhibition of cell-cell adhesion and hypoxia response may disrupt normal cardiomyocyte structure, contributing to the observed cardiac dysfunction. DEPs were predominantly enriched in the cytoplasm, extracellular matrix, and membrane (Fig. 5). Molecular functions related to copper and calcium molecular binding and protein tyrosine kinase activity were significantly enriched (Fig. 5). These functions may play a role in the regulation of ion transport, cell signaling, and stress responses in cardiac tissue.

**Figure 6:**
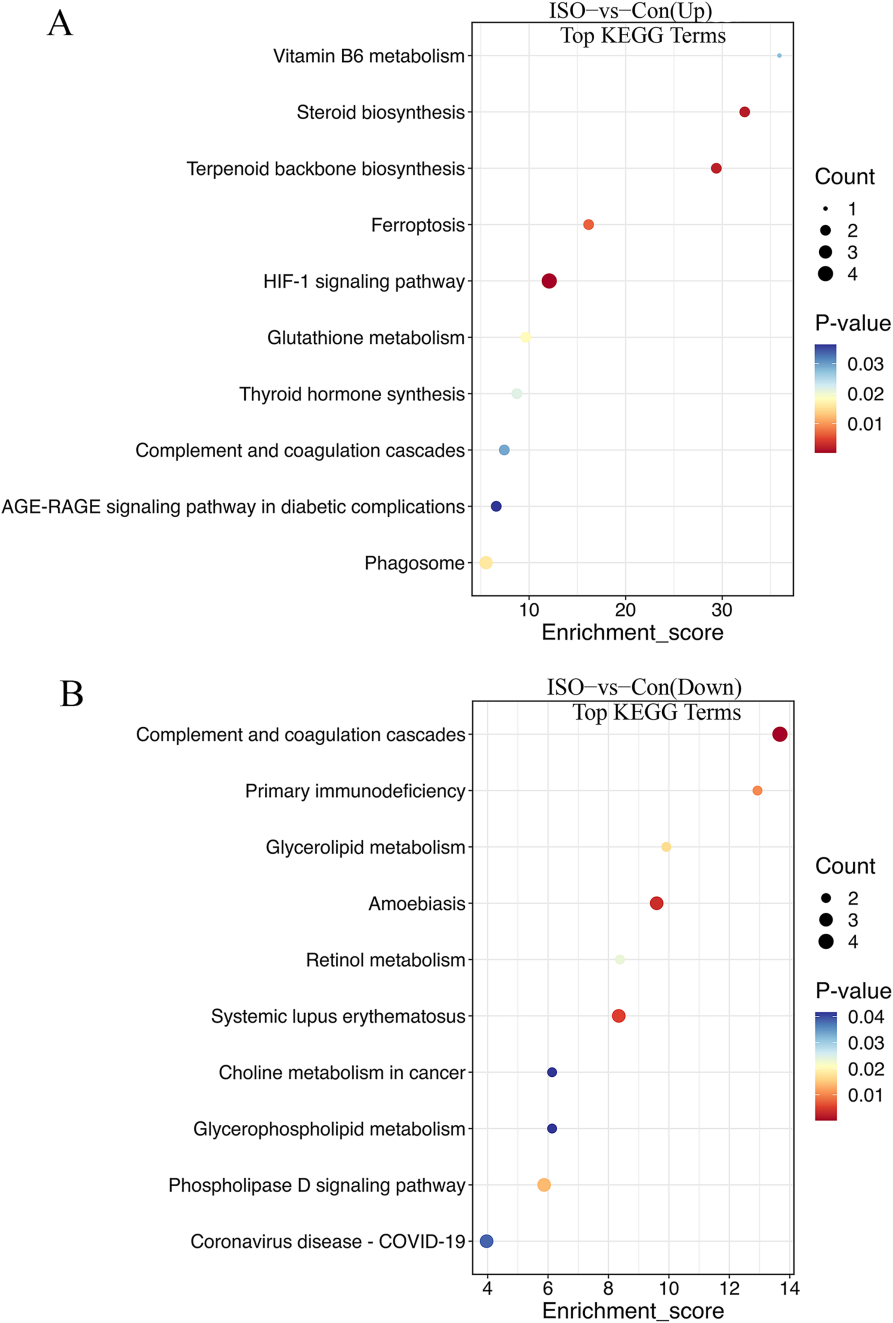
Kyoto Encyclopedia of Genes and Genomes (KEGG) pathway enrichment analysis of DEPs. (A) KEGG analysis of upregulated DEPs shows significant enrichment in pathways such as ferroptosis, glutathione metabolism, and biosynthesis of unsaturated fatty acids. (B) Downregulated DEPs are enriched in pathways including primary immunodeficiency, complement and coagulation cascades, and cholesterol metabolism. Pathways were considered significantly enriched based on *p* < 0.05, with data derived from five biological replicates per group.

The KEGG enrichment analysis provided further insight into the metabolic and signaling pathways altered by ISO-induced TTC: biosynthesis of unsaturated fatty acids, glutathione metabolism, steroid biosynthesis, vitamin B6 metabolism, fructose and mannose metabolism, pentose phosphate pathway, HIF-1 signaling pathway, and ferroptosis (Fig. 6A). These upregulated pathways suggest a link to stress response or metabolic regulation, involving changes in energy homeostasis and management of oxidative stress. DEPs that were downregulated were enriched in pathways including primary immunodeficiency, complement and coagulation cascades, pantothenate and CoA biosynthesis, cholesterol metabolism, glycerophospholipid metabolism, and fat digestion and absorption (Fig. 6B). This downregulation could indicate altered coagulation and inflammatory responses, as well as reduced energy metabolism or fatty acid synthesis, reflecting broader metabolic disruptions following ISO treatment.

### Protein-protein interaction network analysis

**Figure 7:**
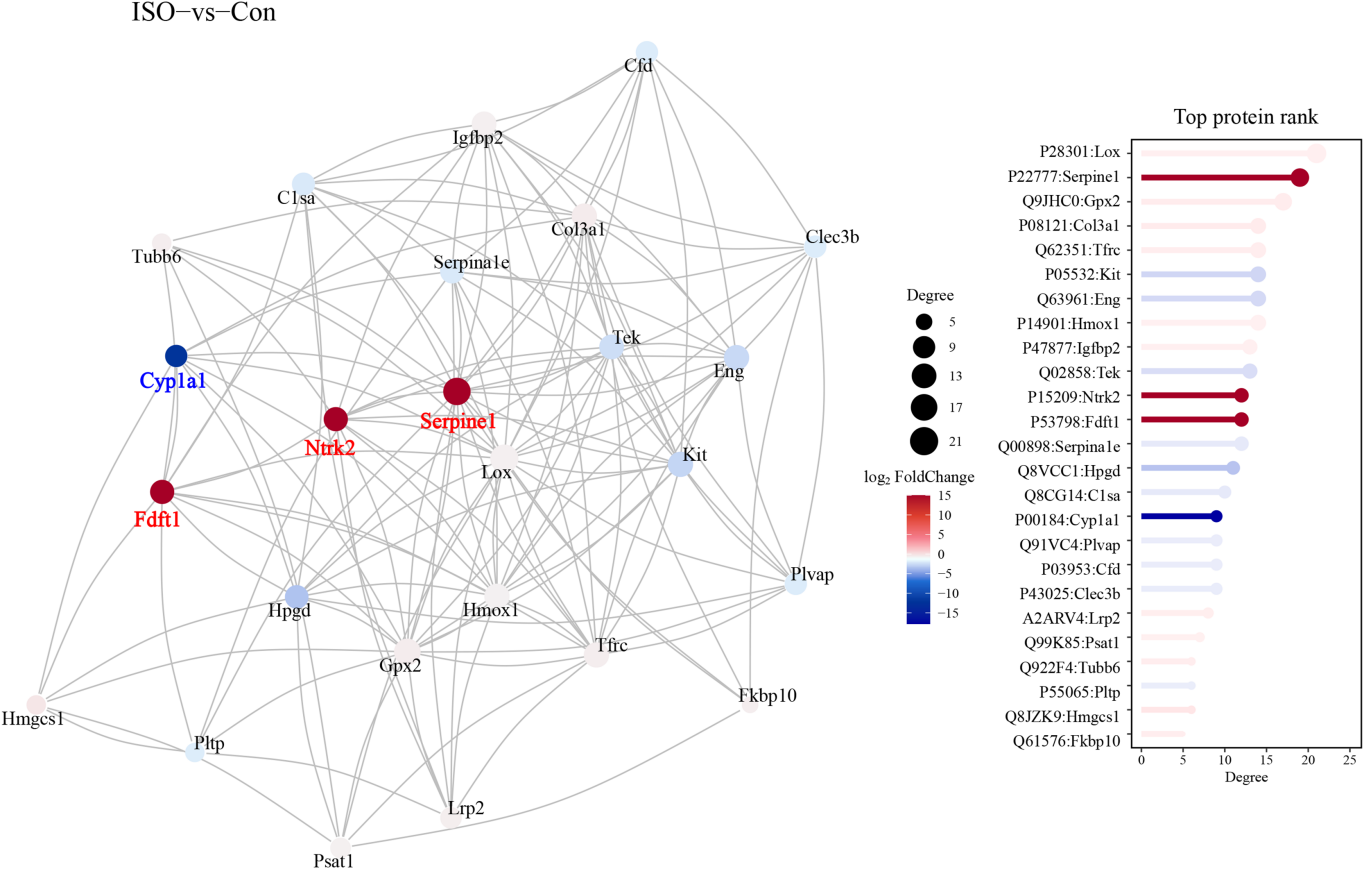
Protein-protein interaction (PPI) network analysis of DEPs. Protein-protein interaction network was constructed using the STRING database, visualized in Cytoscape. Hub proteins were identified based on degree centrality, including Ntrk2, Fdft1, Serpine1, and Cyp1a1. The network highlights key interactions among DEPs, with nodes representing proteins and edges indicating functional associations. Confidence score threshold = 0.7.

A protein-protein interaction (PPI) network was constructed to explore the functional associations between DEPs. This network analysis aimed to identify key regulatory hubs and interactions that may play critical roles in the pathophysiology of stress-induced myocardial injury. The PPI network revealed several key hubs, including Ntrk2, Fdft1, and Serpine1, Cyp1a1 (Fig. 7).

### Gene set enrichment analysis

**Figure 8:**
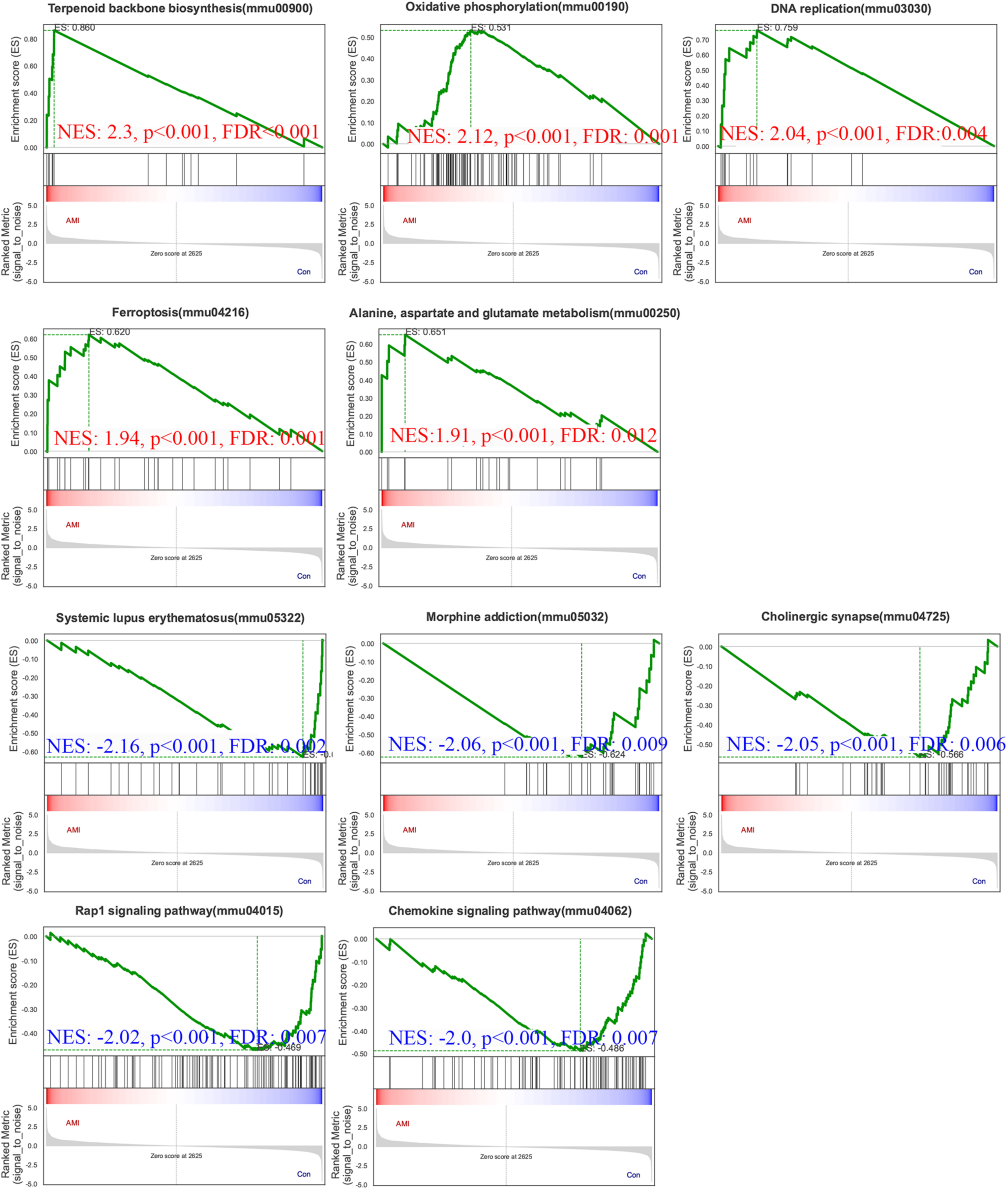
Gene Set Enrichment Analysis (GSEA) of ISO-treated *vs*. control groups. GSEA results reveal significantly enriched upregulated pathways in ISO-treated mice, including oxidative phosphorylation, ferroptosis, and terpenoid backbone biosynthesis (normalized enrichment score [NES] > 1.91, *p* < 0.01). Downregulated pathways include systemic lupus erythematosus, Rap1 signaling, and chemokine signaling (NES < −2.00, *p* < 0.01). Data represent normalized enrichment scores based on five biological replicates per group.

To gain further insights into the biological processes and pathways involved in ISO-induced TTC, we performed GSEA using the KEGG database. Pathways enriched in genes that are highly expressed in the experimental condition compared to the control. A positive normalized enrichment score (NES) indicates that the pathway is enriched at the top of the ranked gene list (upregulated). Biological processes or signaling pathways with high NES values are often associated with the condition’s phenotype, indicating activation of these pathways. The GSEA five significantly enriched upregulated pathways in response to acute stress (Fig. 8): terpenoid backbone biosynthesis (normalized enrichment score; NES = 2.30, *p* = 0.0038), oxidative phosphorylation (NES = 2.12, *p* = 0.0018), DNA replication (NES = 2.04, *p* = 0.0014), ferroptosis (NES = 1.94, *p* = 0.0018), alanine, aspartate and glutamate metabolism (NES = 1.91, *p* = 0.0013).

Pathways enriched in genes with reduced expression in the experimental condition compared to the control. Pathways enriched in genes with reduced expression in the experimental condition compared to the control. Five significantly enriched downregulated pathways were also identified (Fig. 8): systemic lupus erythematosus (NES = −2.16, *p* = 0.0024), morphine addiction (NES = −2.06, *p* = 0.0022), cholinergic synapse (NES = −2.05, *p* = 0.0024), Rap1 signaling pathway (NES = −2.02, *p* = 0.0024), chemokine signaling pathway (NES = −2.00, *p* = 0.0023).

The upregulation of pathways related to energy metabolism, oxidative stress, and ferroptosis points to significant metabolic and oxidative challenges in the heart during acute stress. Conversely, the downregulation of pathways involved in immune responses, cell adhesion, and neurotransmitter signaling reflects broader disruptions in both inflammatory and nervous system regulation, which may further contribute to the progression of stress-induced cardiac dysfunction.

### Dual validation of DEPs using qPCR and western blot

**Figure 9:**
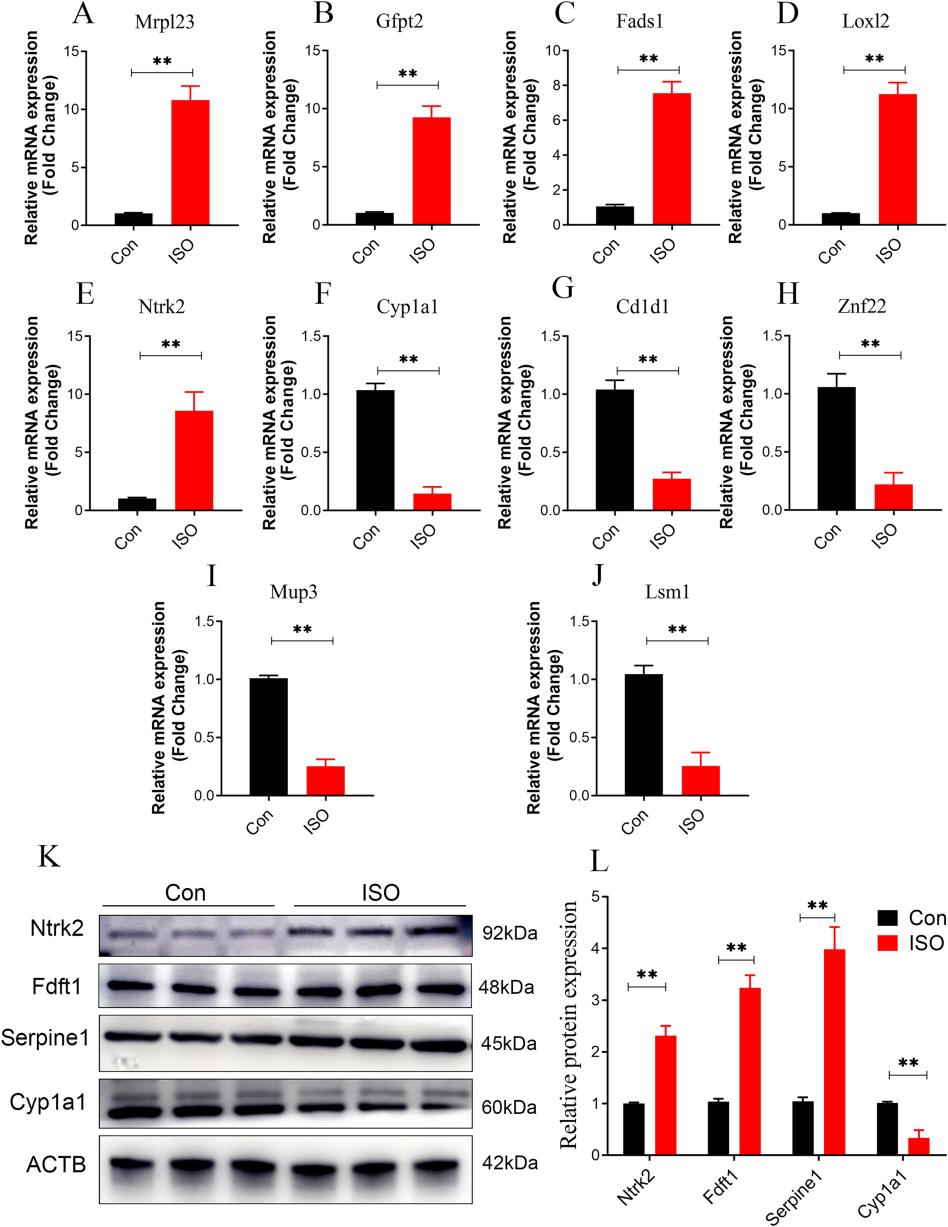
Dual validation of DEPs using qPCR and Western Blot. (A–J) qPCR validation of the top 5 upregulated and top 5 downregulated DEPs, showing consistent expression trends between qPCR and proteomics data. Data represent mean ± SD from 3 biological replicates per group. Statistical significance was determined using Student ’ s t-test (**p < 0.01 compared to control). (K, L) Western Blot validation of key hub proteins (Ntrk2, Fdft1, Serpine1, and Cyp1a1) identified through PPI network analysis. Representative Western Blot images and quantification of protein expression normalized to beta-actin (loading control) are shown. Data represent mean ± SD from three biological replicates per group. Statistical significance was determined using Student’s t-test (**p < 0.01 compared to control).

To further validate the reliability of these findings, qPCR was performed on the top five upregulated and downregulated DEPs (10 genes in total). The qPCR results were highly consistent with the proteomics data, reinforcing the reliability of the observed protein expression patterns (Figs. 9A–9J). Additionally, key hub proteins identified through PPI network analysis, including Ntrk2, Fdft1, Serpine1, and Cyp1a1, were validated using Western Blot (WB). The WB results corroborated the differential expression trends of these hub proteins, further underscoring the robustness of the proteomics analysis (Figs. 9K, 9L).

## Discussion

Takotsubo cardiomyopathy (TTC), which can be triggered by various stressors, exhibits a wide range of clinical presentations and affects different areas of the heart (Ghadri et al., 2018). Although using a Takotsubo-like animal model simplifies the disease’s complexity, it provides a useful platform for conducting rapid and consistent research in a uniform population. This approach allows for repeated longitudinal assessments within the same subject, offering important insights into the natural progression of the disease over time.

Epidemiological studies show that TTC predominantly affects postmenopausal women, with over 80% of cases reported in females (Boland, Lee & Bleck, 2015). This gender disparity is thought to be influenced by differences in sex hormones, specifically the protective role of estrogen (Fu et al., 2021). Estrogen regulates antioxidant defenses and nitric oxide bioavailability, which are critical for maintaining cardiovascular health. In our study, female mice were selected to model the gender-specific susceptibility observed in humans.

In our model, we observed a rapid onset of left ventricular dysfunction, characterized by a reduced LVEF and the typical apical ballooning, closely mirroring the clinical presentation of TTC. This temporary cardiac dysfunction was followed by an almost complete recovery by day 7 post-injection, emphasizing the reversible nature of the heart damage induced by acute catecholamine surges. These findings are consistent with previous studies that reported similar in rodent models treated with high doses of ISO (Xing et al., 2022; Mangali et al., 2021). However, despite the recovery of functional parameters, histological analysis showed that structural changes in the myocardium persisted, suggesting that tissue remodeling and cellular injury may continue beyond the restoration of cardiac function. Histological examination of myocardial tissue from ISO-injected female mice revealed progressive changes in myocardial cell structure over time, highlighting the evolving nature of stress-induced cardiac damage.

### Proteomic insights into stress-induced myocardial damage

To further explore the mechanisms underlying the development of TTC, we conducted a proteomics analysis comparing myocardial tissue from mice 1-day post-ISO injection with that from control mice. Proteomics offers significant advantages in identifying key proteins involved in disease pathogenesis (McArdle & Menikou, 2021). For example, it allows for the comprehensive quantification of protein expression changes, helping us uncover critical proteins that may drive the onset and progression of disease states like TTC (Hanash, 2003).

Our label-free quantitative proteomics analysis identified 81 DEPs, with 39 upregulated and 42 downregulated proteins following ISO treatment. Among the upregulated proteins, several are associated with cellular stress responses and metabolic dysregulation, such as Mrpl23, Gfpt2, and Loxl2, suggesting their involvement in acute stress-induced metabolic shifts and tissue remodeling. Notably, loxl2 is known to play critical roles in extracellular matrix (ECM) organization and fibrosis (Dinca et al., 2021), indicating that ISO-induced myocardial damage may involve early fibrotic changes, which have been corroborated by histological studies that demonstrated structural changes in the myocardium by day 7. Additionally, the downregulation of proteins such as Cyp1a1 and Cd1d1 hints at impaired detoxification and immune regulation in response to ISO treatment. Cyp1a1, in particular, is involved in xenobiotic metabolism (Mescher & Haarmann-Stemmann, 2018), and its downregulation could suggest a reduced ability to detoxify harmful byproducts generated during acute stress, contributing to cardiomyocyte damage.

### Biological significance of ferroptosis and collagen reorganization in myocardial injury

GO and KEGG pathway analyses provided further insights into the biological processes disrupted by ISO-induced stress. GO analysis revealed significant enrichment in pathways related to collagen fibril organization and elastic fiber assembly. The upregulation of pathways such as collagen fibril organization (Sadri et al., 2022) and elastic fiber assembly (Lin, Cocciolone & Wagenseil, 2022) aligns with the observed structural remodeling in the myocardium, supporting the notion that TTC involves not only functional impairment but also early signs of myocardial fibrosis. The downregulation of pathways associated with vascular smooth muscle function, cell adhesion, and hypoxia response further emphasizes the complexity of TTC pathogenesis. For example, the suppression of cell-cell adhesion may weaken myocardial integrity (McFaul & Fernandez-Gonzalez, 2017), while the impaired hypoxia response could exacerbate ischemic injury during acute stress events (Xia et al., 2018). These findings suggest that structural remodeling of the extracellular matrix (ECM) occurs in response to acute stress. Collagen reorganization plays a crucial role in maintaining the structural and mechanical integrity of the myocardium. Abnormal collagen remodeling, however, may impair left ventricular function and contribute to myocardial stiffness, exacerbating the dysfunction observed in TTC. This observation is consistent with previous research linking ECM remodeling to adverse cardiac outcomes.

KEGG analysis highlighted the upregulation of key metabolic pathways, including glutathione metabolism and ferroptosis, indicating that ISO-induced TTC is associated with significant oxidative stress and disrupted metabolic homeostasis. Our findings indicate that ferroptosis and collagen reorganization may play critical roles in ISO-induced TTC. Ferroptosis, a form of regulated cell death driven by iron-dependent lipid peroxidation, was prominently enriched in our analysis. This pathway is closely associated with oxidative stress and mitochondrial dysfunction, which are hallmarks of myocardial injury (Fang et al., 2023). The upregulation of ferroptosis-related pathways, such as glutathione metabolism, suggests an adaptive response by cardiac cells to counteract oxidative damage. However, the persistence of ferroptosis activation may exacerbate cardiac injury by promoting further oxidative damage and compromising cellular homeostasis. This finding aligns with previous studies highlighting ferroptosis as a key mechanism in cardiac conditions such as ischemia-reperfusion injury, suggesting that ferroptosis may also serve as a potential therapeutic target for TTC (Matkarimov & Saparbaev, 2020), potentially as part of a survival mechanism to cope with stress-induced injury.

### Key regulatory proteins and ppi network analysis in ISO-induced TTC

The PPI network analysis identified several key regulatory hubs, including Ntrk2, Serpine1, and Fdft1, which are implicated in distinct yet interconnected biological processes relevant to TTC. Ntrk2, encoding the tropomyosin receptor kinase B (TrkB), plays a central role in neurotrophic signaling (Luningschror & Sendtner, 2024). TrkB is activated by brain-derived neurotrophic factor (BDNF), which is known to mediate neuroprotection, stress adaptation, and cardiac function regulation (Yang et al., 2023). Serpine1 encodes PAI-1, a regulator of fibrinolysis that promotes thrombosis by inhibiting fibrin breakdown (Racine et al., 2024). Fdft1, a key enzyme in cholesterol biosynthesis, may reflect the myocardium’s attempt to repair cell membranes and maintain metabolic homeostasis. However, dysregulated lipid metabolism could exacerbate oxidative stress and inflammation, linking Fdft1 to ferroptosis and myocardial injury (Chen et al., 2023). These suggest that the sympathetic nervous system, fibrinolytic regulation, thrombosis and coagulation pathways might also contribute to the TTC pathology, aligning with the known pro-thrombotic risk in stress-induced cardiomyopathies.

In our study, we observed that the acute myocardial dysfunction induced by ISO gradually improved by day 7 post-injection. This recovery likely reflects the resolution of catecholamine toxicity, which initially causes oxidative stress, calcium overload, and energy metabolism disruption. Once ISO levels decline, the heart may regain homeostasis, allowing for functional recovery. Proteomic analysis further revealed the upregulation of key reparative pathways, including oxidative phosphorylation, glutathione metabolism, and DNA replication, suggesting that mitochondrial restoration, oxidative stress mitigation, and cellular repair processes contribute to recovery. These findings point to several potential therapeutic strategies to enhance myocardial recovery. Antioxidants targeting oxidative stress, ferroptosis inhibitors to prevent lipid peroxidation, and agents modulating ECM remodeling could accelerate the restoration of cardiac function. Additionally, enhancing neurotrophic signaling or providing metabolic support could further improve myocardial adaptation and repair. Future studies are warranted to validate these mechanisms and explore their translational potential for developing targeted therapies to mitigate stress-induced myocardial injury and promote recovery.

## Limitations and future directions

While this study provides valuable insights into the molecular mechanisms of TTC, several limitations should be acknowledged. First, the murine model, though effective in mimicking many clinical features of TTC, does not fully replicate the complexity of human disease. Differences in stress responses, myocardial metabolism, and hormonal regulation, particularly the role of estrogen in postmenopausal women, may affect the generalizability of the findings. Second, proteomic analysis offers a broad overview of protein expression but does not capture post-translational modifications or dynamic protein-protein interactions, which may play critical roles in disease progression. Integrating other omics approaches, such as phosphoproteomics or interactomics, could provide deeper mechanistic insights. Lastly, while key pathways and proteins were identified, their validation in human TTC patients is essential to confirm their relevance. Exploring the therapeutic potential of targeting pathways like ferroptosis, oxidative phosphorylation, and ECM remodeling in both preclinical and clinical settings could offer new strategies to mitigate stress-induced myocardial injury and promote recovery. Despite these limitations, this study lays a strong foundation for understanding TTC and highlights promising avenues for future research and therapeutic development.

## Conclusion

In this study, we explored the pathophysiological mechanisms underlying ISO-induced TTS-like cardiomyopathy in female mice. Our proteomics study provides novel insights into the molecular mechanisms underlying stress-induced myocardial injury. The identification of key proteins involved in oxidative stress, inflammation, and energy metabolism highlights the multifaceted nature of myocardial responses to acute stress. The integration of functional, structural, and proteomics data provides a comprehensive understanding of TTC, paving the way for future studies aimed at developing targeted therapies to mitigate the long-term effects of acute stress on the heart.

## Supplemental Information

10.7717/peerj.18984/supp-1 Supplemental Information 1 The sequences of primers used in this study.

10.7717/peerj.18984/supp-2 Supplemental Information 2 The DEPS between ISO-Con group.

10.7717/peerj.18984/supp-3 Supplemental Information 3 Heatmap of the top 20 DEPs, with hierarchical clustering based on normalized expression levels.

10.7717/peerj.18984/supp-4 Supplemental Information 4 ARRIVE 2.0 checklist.

10.7717/peerj.18984/supp-5 Supplemental Information 5 MIQE checklist.

10.7717/peerj.18984/supp-6 Supplemental Information 6 Raw data.

10.7717/peerj.18984/supp-7 Supplemental Information 7 Original gel images.

## Additional Information and Declarations

### Data Availability

The following information was supplied regarding data availability:

The raw proteomic data analyzed in this study are available at iProX: IPX0009801000.

Raw data are available in the Supplemental Files.
