# Supplementary material for: Proteomics of stress-induced cardiomyopathy: insights from differential expression, protein interaction networks, and functional pathway enrichment in an isoproterenol-induced TTC mouse model
Source: PeerJ. 2025 Feb 13;13:e18984. doi: 10.7717/peerj.18984 (PMC11830371; doi:10.7717/peerj.18984)
Supplement: Supplemental Information 1 [file peerj-13-18984-s001.docx]

| Gene name | Forward primer | Reverse primer |
| --- | --- | --- |
| Mrpl23 | CTATACACCGAGAGGTACCC | GTATCTTCAGGCTGTGCAG |
| Gfpt2 | GATCTAAGGAAGTTTCTGGAAAGC | TTTAATCAGCTTGGCGATGG |
| Fads1 | CTTCATTGTCAGGTTCCTGG | TCGTGATCAATGTGCATGG |
| Loxl2 | ACAACTGTCACGTAGGTGG | GCTGGTTATTTAGAAGTCCACTG |
| Ntrk2 | AGAACGAGTATGGGAAGGA | TTGGGTTTGTCTCGTAGTC |
| Cyp1a1 | GGAGGCCTTCATTCTGGAG | CTTGTATCTCTTGTGGTGCTG |
| Cd1d1 | AATATGTCGTGAGATTCTGGG | CTGGCTTCTCTTGCTTCTC |
| Znf22 | TTGTACGGAAGGGAATTCC | TTTGGGCTTTCCTAACCTC |
| Mup3 | TGCGATTGGTGAACAAACAG | CTGGTTCTCGGCCATAGAG |
| Lsm1 | ACTAGGAGAAATAGACCTGGAG | ACCCTCTGTTCCTCTAGGA |

Supplementary Table 1. The sequences of primers used in this study.
