# Supplementary material for: Proteomics of stress-induced cardiomyopathy: insights from differential expression, protein interaction networks, and functional pathway enrichment in an isoproterenol-induced TTC mouse model
Source: PeerJ. 2025 Feb 13;13:e18984. doi: 10.7717/peerj.18984 (PMC11830371; doi:10.7717/peerj.18984)
Supplement: Supplemental Information 2 [file peerj-13-18984-s002.docx]

Supplementary Table 2. The DEPS between ISO-Con group.

| Gene name | log2FoldChange | Regulation |
| --- | --- | --- |
| Mrpl23 | 15 | Up |
| Gfpt2 | 15 | Up |
| Fads1 | 15 | Up |
| Loxl2 | 15 | Up |
| Ntrk2 | 15 | Up |
| Serpine1 | 15 | Up |
| -- | 15 | Up |
| Fdft1 | 15 | Up |
| Fads3 | 15 | Up |
| Hmgcs1 | 0.9835274 | Up |
| Ahsp | 0.9665476 | Up |
| Lss | 0.9260202 | Up |
| Aldoc | 0.9087814 | Up |
| Sult1a1 | 0.8315264 | Up |
| Col3a1 | 0.7977324 | Up |
| Rrm1 | 0.784952 | Up |
| Filip1l | 0.774516 | Up |
| Gpx2 | 0.7596642 | Up |
| Sorcs2 | 0.7569258 | Up |
| Cirbp | 0.7470118 | Up |
| Rbm3 | 0.7375188 | Up |
| Tbce | 0.7268648 | Up |
| Fbxo3 | 0.715858 | Up |
| Tubb6 | 0.7046396 | Up |
| Tfrc | 0.6999104 | Up |
| Fkbp10 | 0.6987282 | Up |
| Lrp2 | 0.6849378 | Up |
| Dysf | 0.6487428 | Up |
| Samd9l | 0.6480644 | Up |
| Lox | 0.6318906 | Up |
| Crtap | 0.6274256 | Up |
| Tmlhe | 0.6210692 | Up |
| Igfbp2 | 0.6174636 | Up |
| Mvk | 0.6133158 | Up |
| Kpna2 | 0.6125538 | Up |
| Psat1 | 0.607134 | Up |
| Mcm5 | 0.602731 | Up |
| Mbl2 | 0.5959204 | Up |
| Hmox1 | 0.5860212 | Up |
| She | -0.5856742 | Down |
| Pltp | -0.5870208 | Down |
| Reck | -0.5881702 | Down |
| Ece1 | -0.5993606 | Down |
| Cfd | -0.6048492 | Down |
| Plvap | -0.6054574 | Down |
| Antxr2 | -0.6137376 | Down |
| Clec3b | -0.6207744 | Down |
| Myzap | -0.6212958 | Down |
| Dgkz | -0.6362556 | Down |
| H3-5 | -0.6448158 | Down |
| Kank4 | -0.6468482 | Down |
| Dhrs3 | -0.6517328 | Down |
| Mcf2l | -0.6547584 | Down |
| Hfe | -0.6601844 | Down |
| Plpp3 | -0.6866346 | Down |
| Kiaa1324 | -0.6986362 | Down |
| Serpina1e | -0.706599 | Down |
| Znf512 | -0.7078264 | Down |
| C1sa | -0.7176356 | Down |
| C9 | -0.7361818 | Down |
| Naalad2 | -0.7698992 | Down |
| Zbtb20 | -0.7895614 | Down |
| Vsnl1 | -0.8667972 | Down |
| Ntn1 | -0.9060268 | Down |
| Vnn1 | -0.923796 | Down |
| -- | -1.0583794 | Down |
| Gzma | -1.0771296 | Down |
| Tek | -1.0809418 | Down |
| Eng | -1.2176178 | Down |
| Mup18 | -1.2433264 | Down |
| Kit | -1.3309874 | Down |
| Lifr | -1.4735878 | Down |
| Iglc2 | -1.601219 | Down |
| Mup20 | -1.8271308 | Down |
| Hpgd | -1.9601136 | Down |
| Cyp1a1 | -15 | Down |
| Cd1d1 | -15 | Down |
| Znf22 | -15 | Down |
| Mup3 | -15 | Down |
| Lsm1 | -15 | Down |
| Col4a4 | -15 | Down |
