# Supplementary figures and images for: Proteomics of stress-induced cardiomyopathy: insights from differential expression, protein interaction networks, and functional pathway enrichment in an isoproterenol-induced TTC mouse model
Source: PeerJ. 2025 Feb 13;13:e18984. doi: 10.7717/peerj.18984 (PMC11830371; doi:10.7717/peerj.18984)

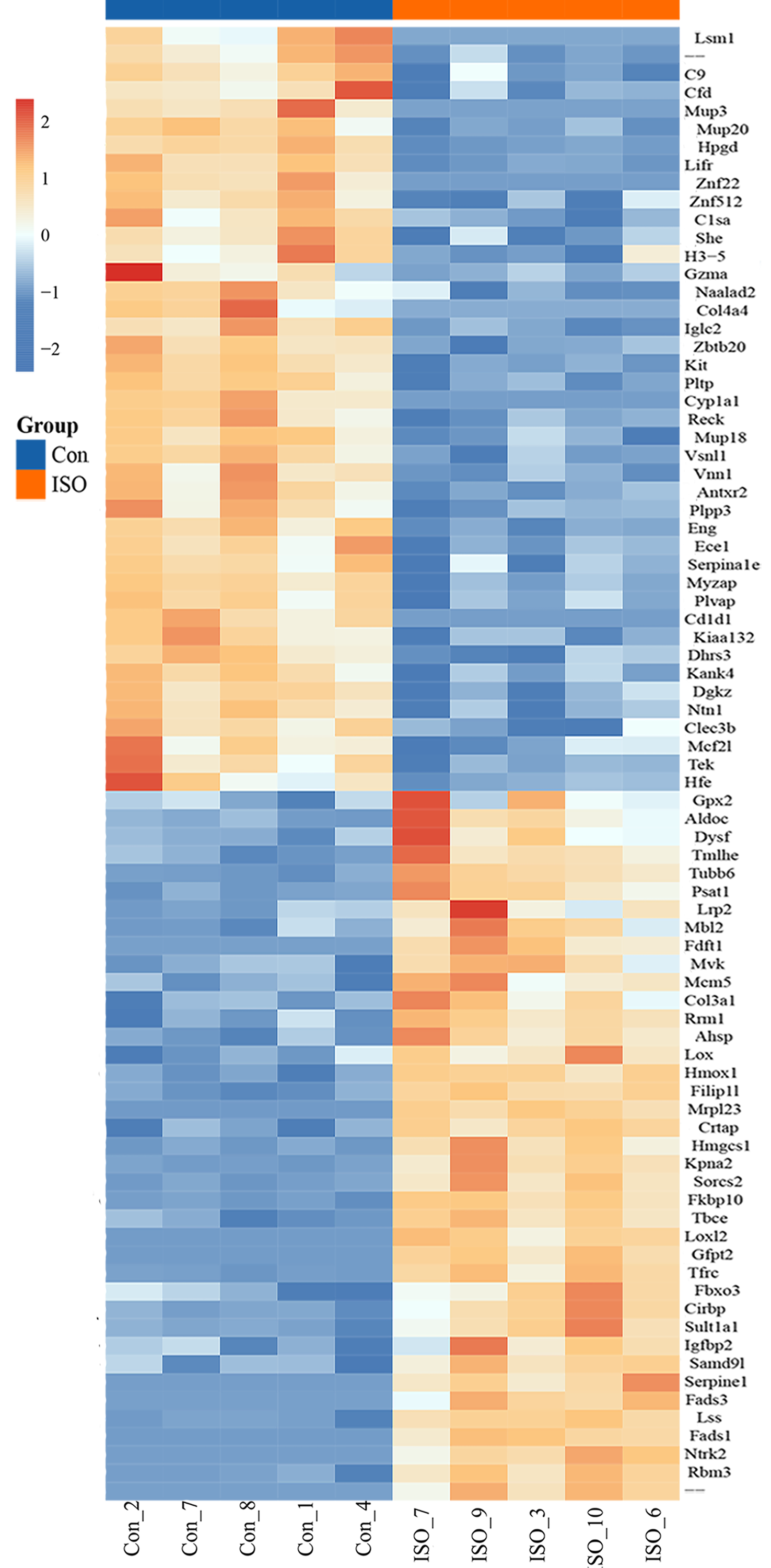

Supplement: Supplemental Information 3 [file peerj-13-18984-s003.png]
