# Supplementary material for: Proteomics of stress-induced cardiomyopathy: insights from differential expression, protein interaction networks, and functional pathway enrichment in an isoproterenol-induced TTC mouse model
Source: PeerJ. 2025 Feb 13;13:e18984. doi: 10.7717/peerj.18984 (PMC11830371; doi:10.7717/peerj.18984)
Supplement: Supplemental Information 7 [file peerj-13-18984-s007.pdf]

Nrtk2 (92kDa)

180kDa
135kDa
98kDa
75kDa

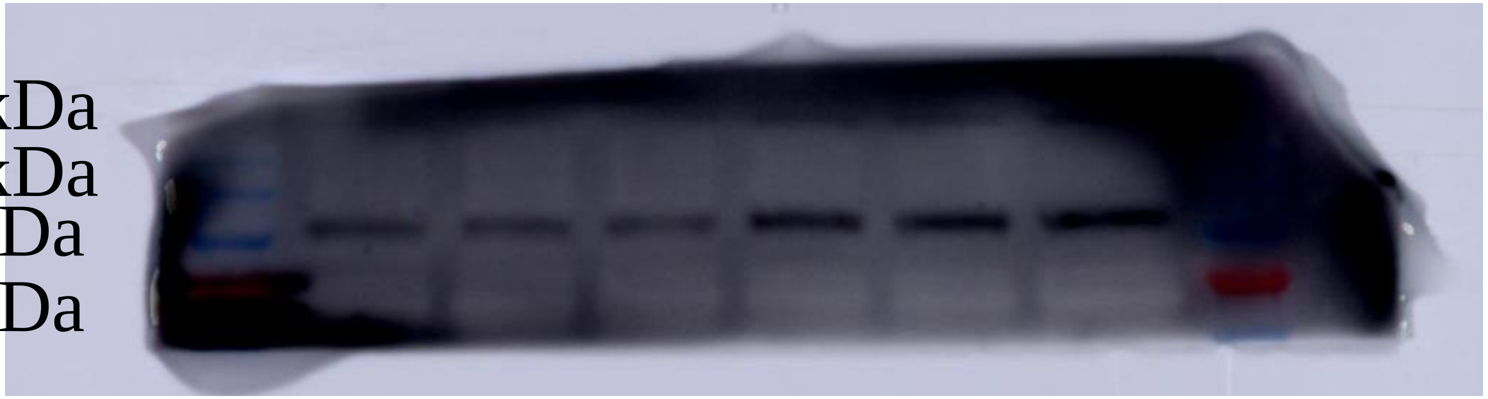

Fdft1 (48kDa)

62kDa
48kDa
34kDa

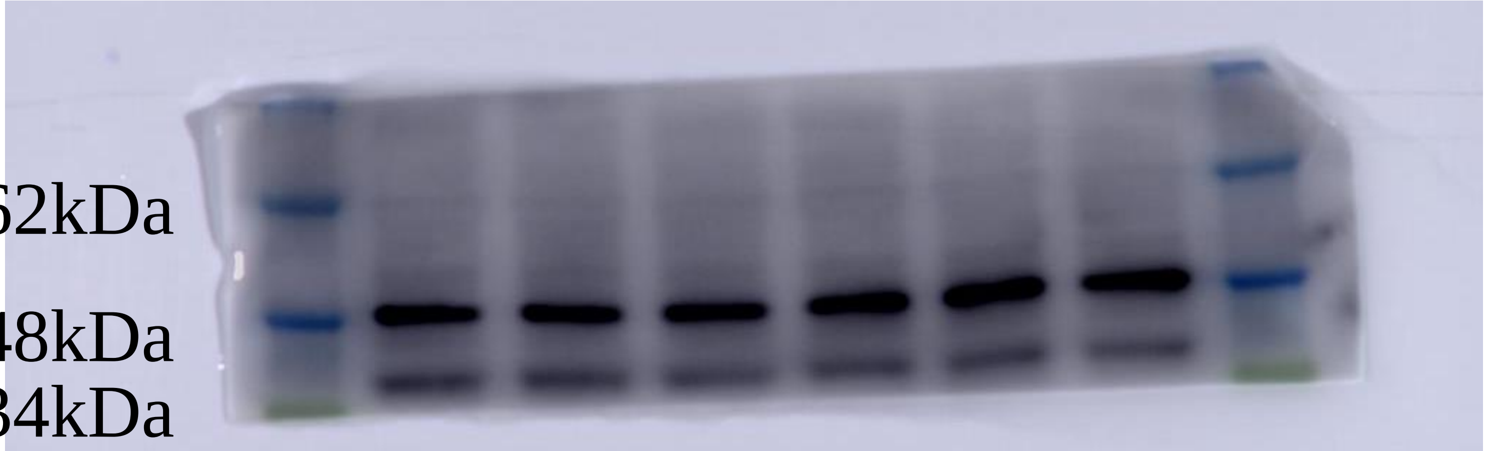

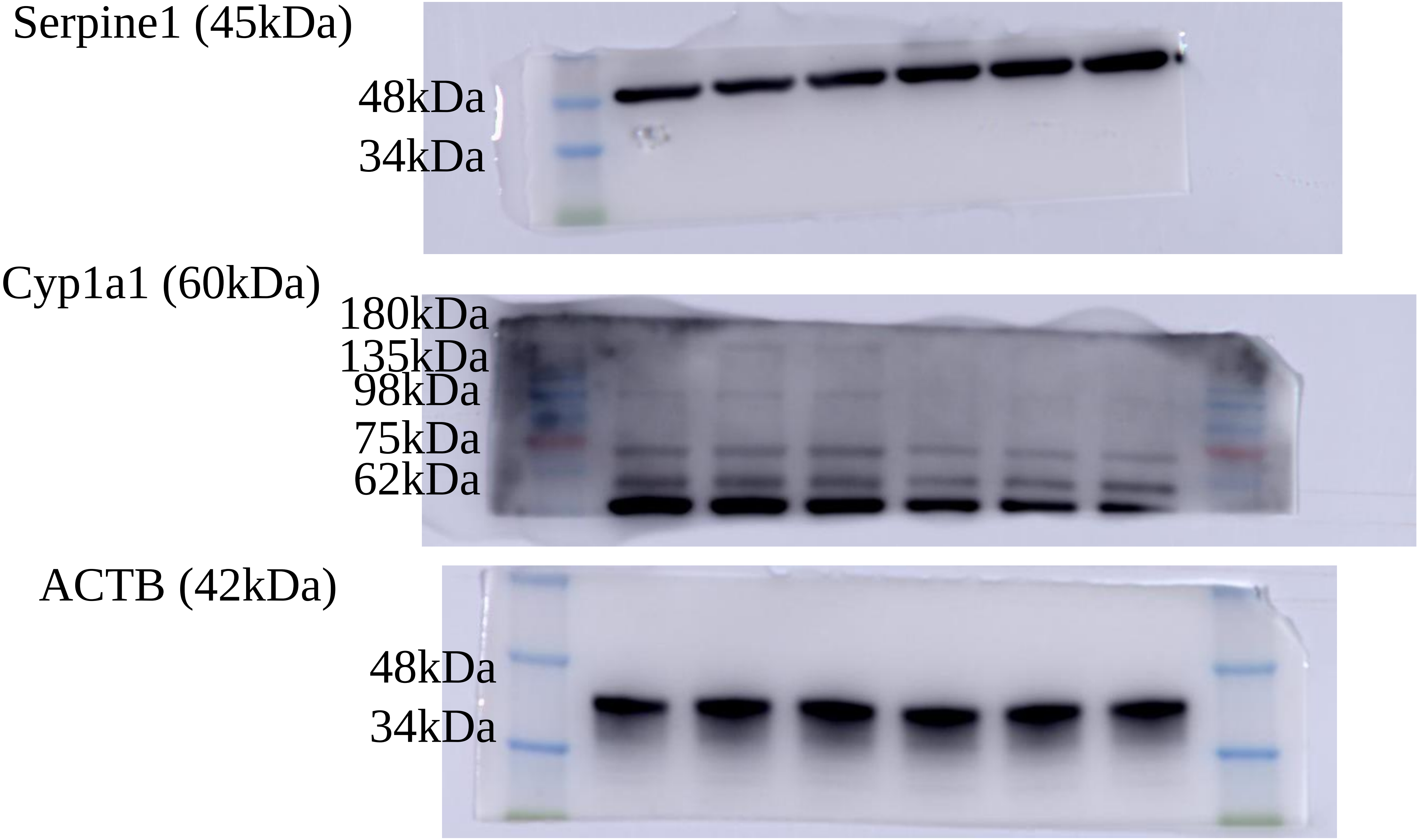
Serpine1 (45kDa)
48kDa
34kDa
Cyp1a1 (60kDa)
180kDa
135kDa
98kDa
75kDa
62kDa
ACTB (42kDa)
48kDa
34kDa
